# *Pseudozyma aphidis* activates reactive oxygen species production, programmed cell death and morphological alterations in the necrotrophic fungus *Botrytis cinerea*

**DOI:** 10.1111/mpp.12775

**Published:** 2019-02-18

**Authors:** Claudia E. Calderón, Neta Rotem, Raviv Harris, David Vela‐Corcía, Maggie Levy

**Affiliations:** ^1^ Department of Plant Pathology and Microbiology, The Robert H. Smith Faculty of Agriculture, Food and Environment The Hebrew University of Jerusalem Rehovot 76100 Israel

**Keywords:** antifungal compound, biocontrol, *Botrytis cinerea*, mode of action, *Pseudozyma aphidis*

## Abstract

Many types of yeast have been studied in the last few years as potential biocontrol agents against different phytopathogenic fungi. Their ability to control plant diseases is mainly through combined modes of action. Among them, antibiosis, competition for nutrients and niches, induction of systemic resistance in plants and mycoparasitism have been the most studied. In previous work, we have established that the epiphytic yeast *Pseudozyma aphidis* inhibits *Botrytis cinerea* through induced resistance and antibiosis. Here, we demonstrate that *P. aphidis* adheres to *B. cinerea* hyphae and competes with them for nutrients. We further show that the secreted antifungal compounds activate the production of reactive oxygen species and programmed cell death in *B. cinerea* mycelium. Finally, *P. aphidis* and its secreted compounds negatively affect *B. cinerea* hyphae, leading to morphological alterations, including hyphal curliness, vacuolization and branching, which presumably affects the colonization ability and infectivity of *B. cinerea*. This study demonstrates additional modes of action for *P. aphidis* and its antifungal compounds against the plant pathogen *B. cinerea*.

## Introduction

*Botrytis cinerea* is a necrotrophic plant pathogen with a broad range of more than 200 plant hosts, which causes significant economic damage to crops worldwide (Williamson *et al.*, 2007), including agriculturally important crops (Elad *et al.*, 2007). Traditionally, *B. cinerea* control has consisted of the repeated use of chemical fungicides. However, this method has become useless owing to the rapid development of resistance (Rupp *et al.*, 2016). The implementation of new strategies to control this pathogen is therefore of high priority. Biological control offers an environmentally friendly alternative to chemical pesticides, and is an attractive means of protecting plants against different phytopathogenic fungi.

Biological control with microbial antagonists has emerged as a promising alternative, with a low environmental impact, either alone or as part of an integrated pest management programme to reduce synthetic fungicide application (Droby *et al.*, 2009; Wilson and Wisniewski, 1994). Among the potential antagonistic strains, yeasts and yeast‐like organisms have been extensively studied as biocontrol agents (Avis and Belanger, 2002; Hammami *et al.*, 2011; Ocampo‐Suarez *et al.*, 2017; Ribes *et al.*, 2018; Santos *et al.*, 2004). This is because yeasts and yeast‐like organisms have simple nutritional requirements, survive in adverse environmental conditions, show good performance against a wide range of pathogens on different commodities (Vardanyan and Hruby, 2016) and are compatible with commercial processing procedures (Droby *et al.*, 2009; Zhimo *et al.*, 2014).

Numerous studies have suggested different antagonistic modes of action of yeasts and yeast‐like organisms against fungi, which seem to be related to the following: the production of diffusible and volatile antifungal metabolites (Avis and Belanger, 2001; Spadaro and Droby, 2016); the induction of defence‐related proteins attributed to the metabolism of proteins, defence response, transcription, energy metabolism and cell structure (Chan *et al.*, 2007); competition for space, nutrition and iron (Bencheqroun *et al.*, 2007; Castoria *et al.*, 2001; Droby *et al.*, 1997; Filonow *et al.*, 1996; Hammami *et al.*, 2011; Janisiewicz *et al.*, 2000; Lima *et al.*, 1997; Liu *et al.*, 2013; Sipiczki, 2006); the promotion of plant growth (Ignatova *et al.*, 2015); the production of glucanase, chitinase, protease and extracellular proteases (Castoria *et al.*, 2001), as well as antifungal hydrolases (El Ghaouth *et al.*, 2003a, b); tolerance to reactive oxygen species (ROS) (Liu *et al.*, 2012); induction of ROS production in the host (Macarisin *et al.*, 2010); the formation of a biofilm (Parafati *et al.*, 2015); and mycoparasitism (Hammami *et al.*, 2011; Henninger and Windisch, 1975; Spadaro and Droby, 2016).

The epiphytic yeast‐like fungus* Pseudozyma aphidis* isolate L12 has been proposed as a biocontrol agent against plant diseases (Barda *et al.*, 2014; Buxdorf *et al.*, 2013a, b; Gafni *et al.*, 2015). A previous report has demonstrated antagonistic activity against *B. cinerea* colonization and spread on tomato (*Solanum lycopersicum*) and *Arabidopsis thaliana* plants, where the biocontrol effect was based on a dual mode of action: antibiosis and induced resistance (Buxdorf *et al.*, 2013a, b). The induced resistance was found to be independent of both SA/NPR‐1 (salicylic acid/ NONEXPRESSOR OF PATHOGENESIS‐RELATED GENE 1) and JAR1/EIN2 (JASMONATE RESISTANT 1/ETHYLENE INSENSITIVE 2) (Buxdorf *et al.*, 2013a, b). Its potential as a biocontrol agent against the cucurbit powdery mildew pathogen *Podosphaera xanthii* via parasitism and antibiosis was demonstrated on cucumber plants (Gafni *et al.*, 2015). Furthermore, *P. aphidis* was found to antagonize the bacterial pathogen *Clavibacter michiganensis* in tomato plants by SA‐independent induced resistance and growth enhancement (Barda *et al.*, 2014).

In this study, we further characterize the modes of action of antagonistic *P. aphidis* L12 during interaction with the pathogenic fungus *B. cinerea*, and the mechanisms by which its antifungal compounds act. We demonstrate the ability of *P. aphidis* to adhere to *B. cinerea* cells and compete with them for nutrient and space. We further show that its secreted antibiotics cause morphological alterations, ROS accumulation and the activation of programmed cell death (PCD) in *B. cinerea* hyphae.

## Results

### Persistence and colonization patterns on cucumber leaves

**Figure 1:**
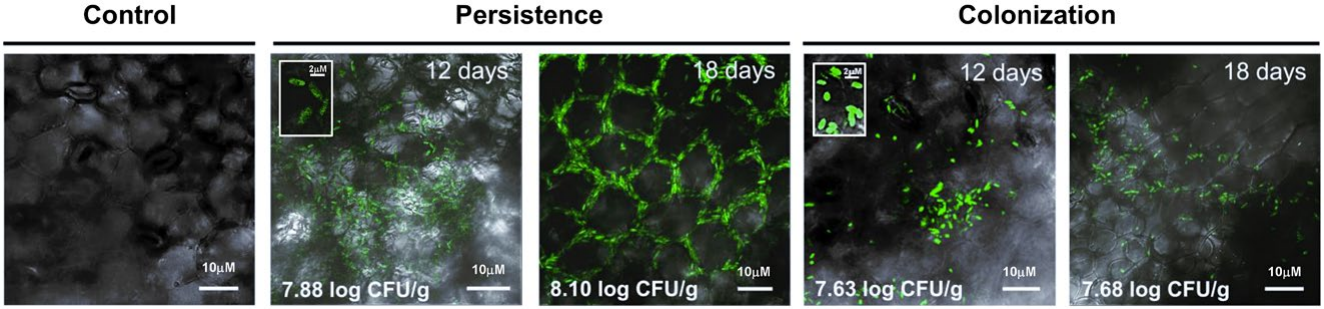
Persistence and colonization of *Pseudozyma aphidis *on cucumber leaves. Two‐week‐old cucumber plants were inoculated with green fluorescent protein (GFP)‐tagged *P. aphidis *and analysed by confocal microscopy. Cells were counted at 12 and 18 days after inoculation. *Pseudozyma aphidis *present on treated leaves was considered to be persistent, and that on new leaves was considered to be a colonizer. Cell counts are presented per gram of tissue at the bottom of the photographs. CFU, colony‐forming units. The insets on the 12 days panels demonstrate yeast‐like morphology.

Persistence and colonization on healthy cucumber plants under high humidity were studied using green fluorescent protein (GFP)‐labelled *P. aphidis*. *Pseudozyma aphidis* cells located on the initial leaves were considered to be persistent, and those present on new leaves were considered to be colonizing. The distribution of *P. aphidis* at 12 days after inoculation was very similar for persistent and colonizing cells, including mainly cells of microcolonies dispersed over the leaf surface (Fig. 1). After 18 days, persistence patterns were characterized by the precise distribution of all of the cells along the junctions of the leaf epidermal cells, whereas colonization patterns included cells along these junctions and in microcolonies. A significantly higher abundance on the leaf surface was observed for the persistent cells [8.10 log colony‐forming units (CFU)/g] than colonizers (7.68 log CFU/g) after 18 days (*t*‐test, *P* < 0.05). We could not observe any tissue damage on healthy plant tissue by persistent or colonizing *P. aphidis*.

### *Pseudozyma aphidis* competes with *B. cinerea* for space and nutrients

To study competition for space and nutrients between *P. aphidis* and *B. cinerea*, an antagonistic experiment using sugar solution on wounded tomato fruit was carried out. First, we characterized the carbon assimilation requirements of our isolate compared with other available *P. aphidis* isolates. As demonstrated in Table 1, our isolate (L12, Israel) was very similar to others (CBS 517.83, IHEM 18822 and DSM 70725) in its ability to grow on different carbon sources. However, on some of the media, there were minor, but clearly visible, differences in colony density amongst the isolates. Noticeably, L12 was the only isolate that was unable to grow on media containing glycerol, erythritol or potassium gluconate as the carbon source, and the only isolate that grew on a medium containing l‐sorbose as the carbon source. Accordingly, we prepared a sugar solution containing d‐galactose, sucrose, l‐arabinose, inositol and d‐glucose for the competition assays on tomato fruit.

**Table 1:** Characterization of *Pseudozyma aphidis* carbon assimilation requirements.

Substrate	L12	CBS 517.83	IHEM 18822	DSM 70725
d‐Galactose	+	+	+	+
Cycloheximide (actidione)	–	–	–	–
d‐Saccharose (sucrose)	+	+	+	+
*N*‐Acetylglucosamine	+	+	+	+
Lactic acid	W	W	W	W
l‐Arabinose	+	+	+	+
d‐Cellobiose	–	–	–	–
d‐Raffinose	+	+	+	+
d‐Maltose	+	+	+	+
d‐Trehalose	W	W	–	W
Potassium 2‐ketogluconate	W	+	W	+
Methyl‐αd‐glucopyranoside	W	W	W	+
d‐Mannitol	W	W	W	W
d‐Lactose (bovine origin)	+	W	+	+
Inositol	W	+	W	W
d‐Sorbitol	+	W	+	+
d‐Xylose	+	+	+	+
d‐Ribose	W	W	W	W
Glycerol	–	W	W	W
l‐Rhamnose	–	–	–	W
Palatinose	+	+	+	+
Erythritol	–	W	W	W
d‐Melibiose	+	+	+	+
Sodium glucuronate	+	+	W	+
d‐Melezitose	+	+	+	+
Potassium gluconate	–	W	W	W
Laevulinic acid (laevulinate)	–	–	–	–
d‐Glucose	+	+	+	+
l‐Sorbose	W	–	–	–
Glucosamine	–	W	–	–

+, positive; W, weakly positive; –, negative (*n* = 3); L12, *P. aphidis* isolated in Israel, 2004.

**Figure 2:**
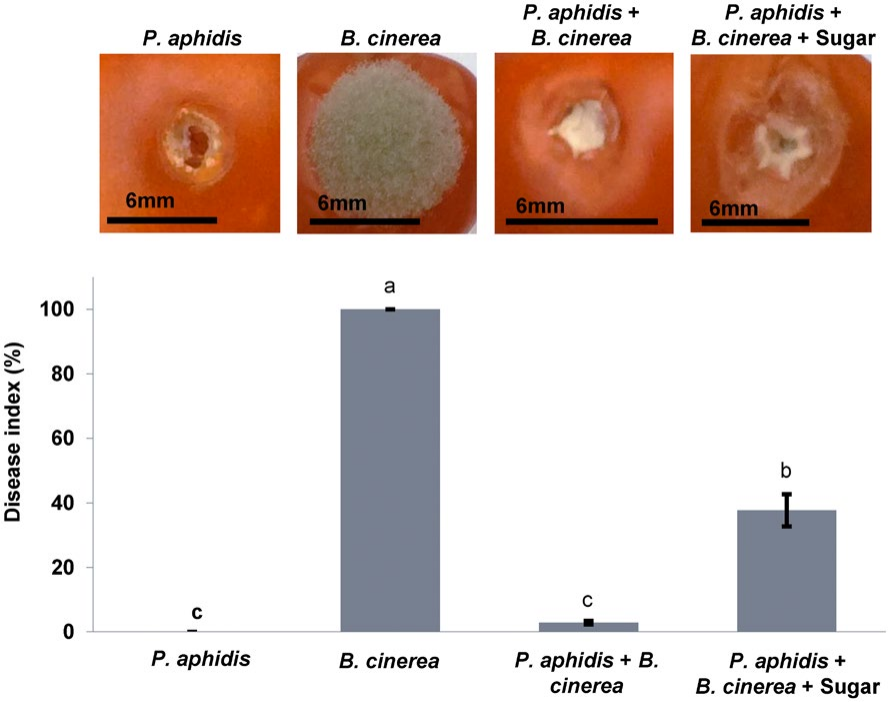
Competition for nutrients. Antagonistic activity of *Pseudozyma aphidis *against *Botrytis cinerea* was assessed on tomato fruit with and without sugar amendment. Inoculated tomatoes were incubated for 6 days at 25 °C in the dark and 95% relative humidity, and the disease index was calculated. Values with different letters denote a statistically significant difference (*P < *0.05, Tukey’s test). Bars, 6 mm for all panels.

Wounded tomatoes treated with *B. cinerea* showed a disease index of 100%, whereas those treated with *P. aphidis*, or *P. aphidis* and *B. cinerea*, exhibited no disease symptoms or a disease index of 2.9%, respectively. However, the antagonistic activity of *P. aphidis* against *B. cinerea* was significantly reduced by the presence of sugar solution. We obtained a 34.8% increase in the *B. cinerea* disease index in tomato wounds treated with *P. aphidis* and supplemented with sugar solution (Fig. 2).

### *Pseudozyma aphidis* secretes lytic enzymes

**Figure 3:**
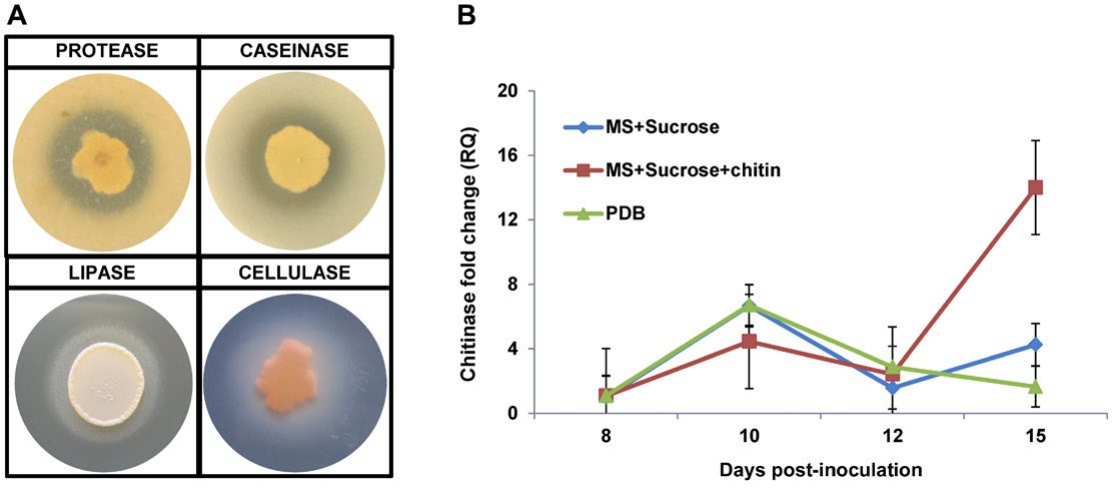
Degrading enzyme activity in *Pseudozyma aphidis*. (A) Protease, caseinase, lipase and cellulase activities were tested in wild‐type *P. aphidis* strain on agar plates. (B) *Pseudozyma aphidis* chitinase expression was measured in different media [Murashige and Skoog (MS) basal medium with sucrose, MS basal medium with sucrose and chitin, and potato dextrose broth (PDB) medium] at 8, 10, 12 and 15 days after inoculation, relative to the expression in MS basal medium without sucrose.

As part of their ability to compete for nutrients and to parasitize pathogens, biological agents secrete lytic enzymes and proteases. The ability of *P. aphidis* to secrete lytic enzymes and proteases was studied on different specifically prepared media. We detected protease, caseinase, lipase and cellulase activity in *P. aphidis* cultures using these media (Fig. 3A).

### *Botrytis cinerea* cell wall extract activates chitinase expression in *P. aphidis*

**Figure 4:**
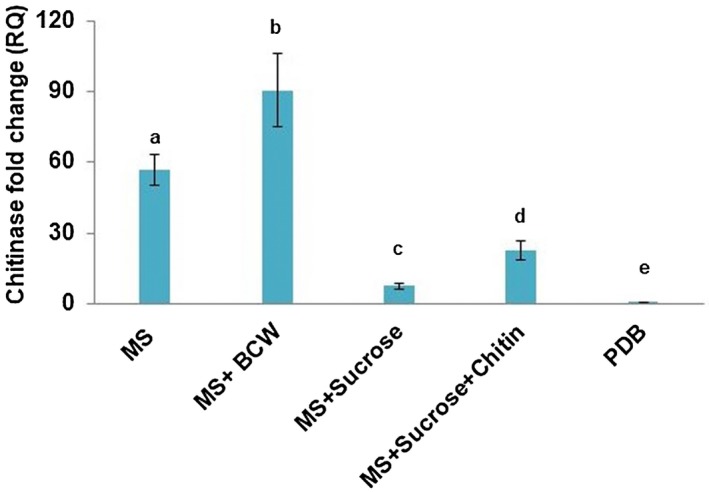
Chitinase expression level in *Pseudozyma aphidis*. *Pseudozyma aphidis* was grown in different media and chitinase expression was measured by quantitative reverse transcription‐polymerase chain reaction (qRT‐PCR) relative to growth in potato dextrose broth (PDB) at 10 days after inoculation. The media were as follows: Murashige and Skoog basal medium (MS); MS with 10 mg of *B. cinerea* cell wall (BCW) suspension; MS with sucrose; MS with sucrose and chitin; and PDB medium as the control. Values with different letters denote a statistically significant difference (*P* < 0.05, Tukey’s test).

To study the ability to utilize the pathogen’s chitin, *P. aphidis* chitinase activity was assessed on different media. As demonstrated in Fig. 3B, we detected higher expression in poor media, such as Murashige and Skoog (MS) basal medium, relative to rich potato dextrose broth (PDB) medium. We also detected the up‐regulation of chitinase when MS basal medium was supplemented with *B. cinerea* cell wall suspension, whereas down‐regulation of chitinase was obtained when MS basal medium was supplemented with sucrose or sucrose and colloidal chitin (Fig. 4).

### *Pseudozyma aphidis* adheres to and alters *B. cinerea* hyphae morphologically and structurally

**Figure 5:**
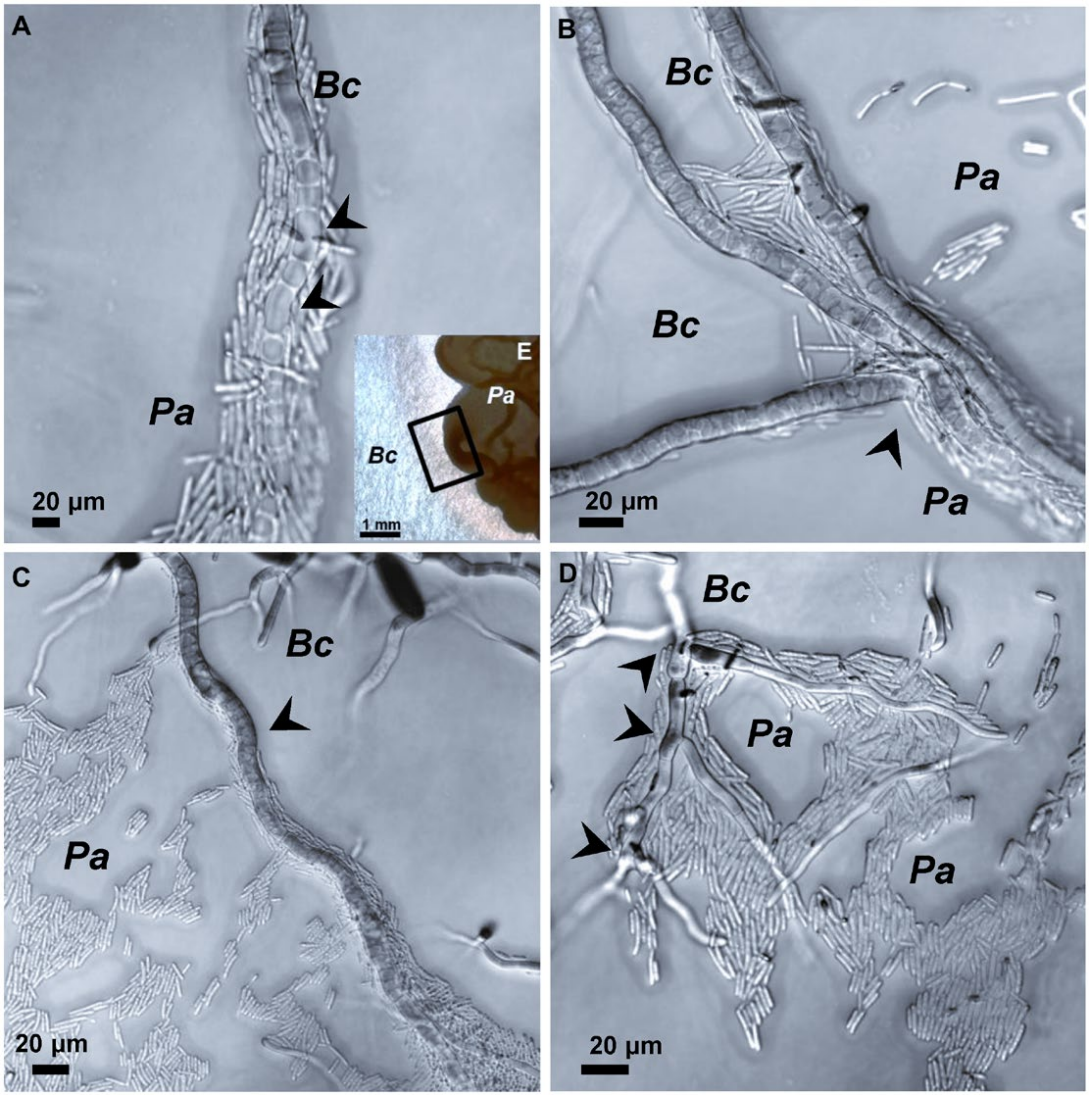
Adhesion of *Pseudozyma aphidis* (*Pa*) to, and morphological alterations in, *Botrytis cinerea* (*Bc*) hyphae during their interaction*. *A dual‐culture test analysed by differential interference contrast (DIC) microscopy demonstrated the adhesion of *P. aphidis* cells to *B. cinerea* hyphae. Hyphal alterations (indicated by arrowheads) included: (A) increased number of vacuoles, (B) changes in the direction of growth, (C) curly growth and (D) branching to resemble a fork‐like structure. (E) Inset zooms in on the interaction area between *B. cinerea *and *P. aphidis*. Bars: (A–D) 20 μm; (E) 1 mm.

A dual‐culture assay was carried out to observe the direct interactions between *P. aphidis* and *B. cinerea*. At the interaction zone (Fig. 5E), *P. aphidis* cells adhered to *B. cinerea* hyphae, covering them and forming complex aggregates (Fig. 5). We also observed morphological alterations in *B. cinerea* hyphae after exposure to *P. aphidis*, such as an increase in vacuole number (Fig. 5A), changes in the direction of hyphal growth (Fig. 5B), curliness (Fig. 5C) and branching (Fig. 5D).

**Figure 6:**
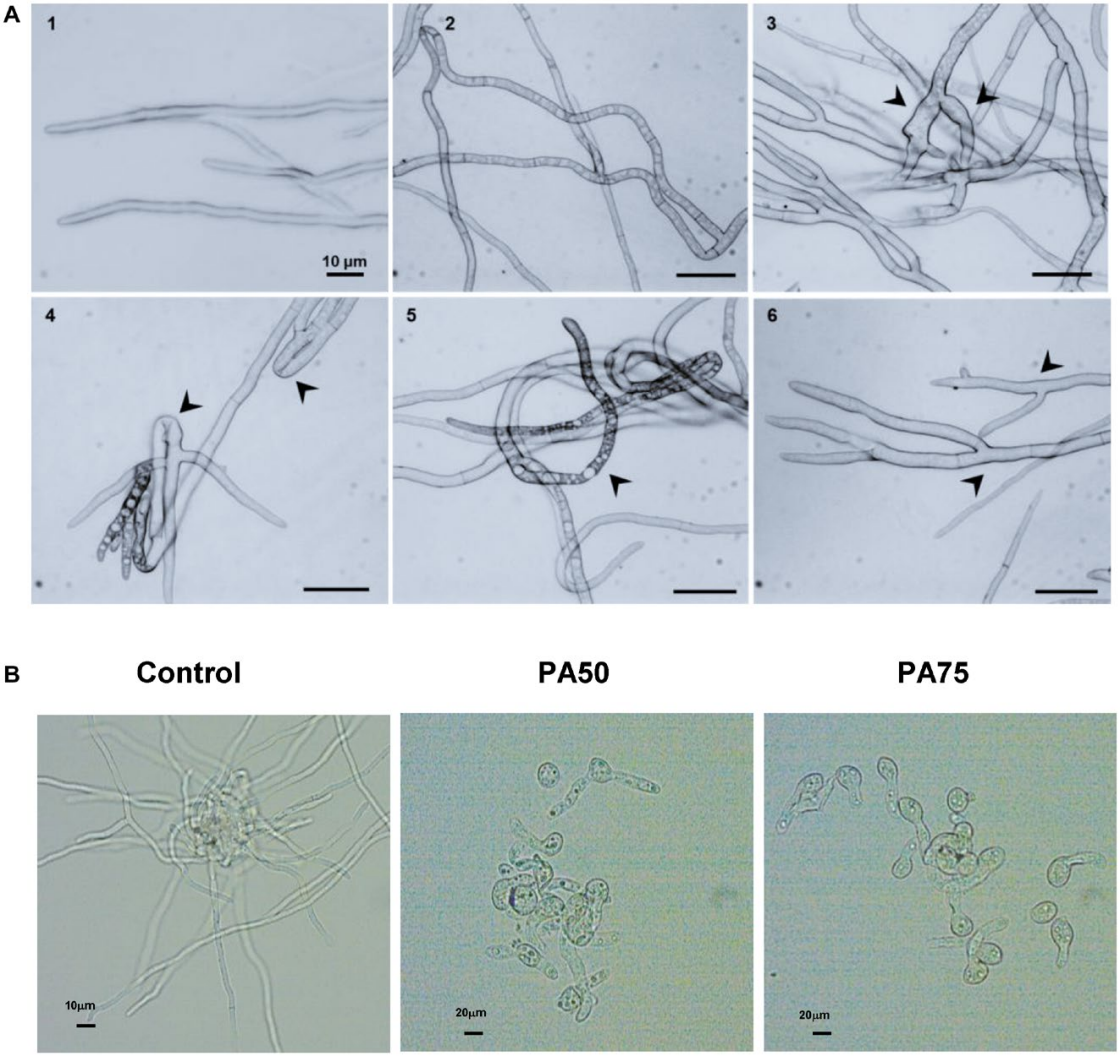
*Pseudozyma aphidis *secretions negatively affect *Botrytis cinerea. *(A) *Botrytis cinerea* hyphal growth alterations after exposure to *P. aphidis* secretions. *Botrytis cinerea* hyphal growth was examined by differential interference contrast (DIC) microscopy after exposure to *P. aphidis* secretions. (A1) Growth of *B. cinerea* in the absence of *P. aphidis *secretions. Exposure to *P. aphidis* secretions causes (A2) hyphal curling, (A3) increased thickness of part of the hyphae, (A4) directional growth disturbance, (A5) increase in vacuole number and (A6) high frequency of hyphal branching close to the hyphal tip. (B) *Botrytis cinerea* spore germination after exposure to 50% (PA50) and 75% (PA75) of *P. aphidis* secretions. Arrowheads point to the different alterations in *B. cinerea *hyphal morphology.

Closer observation under a differential interference contrast (DIC) microscope of the inhibited mycelium after exposure to *P. aphidis* secretions further demonstrated the morphological alterations of *B. cinerea* hyphae. The main alterations in the hyphae were curly growth (Fig. 6A2), altered thickness (Fig. 6A3), directional growth disturbances (Fig. 6A4), an increase in the number of vacuoles (Fig. 6A5) and highly frequent branching close to the hyphal tip (Fig. 6A6). Furthermore, *P. aphidis* secretions trigger strong inhibition of *B. cinerea* spore germination, leading to a reduced hyphal network (Fig. 6B).

### *Pseudozyma aphidis* secretion causes ROS production and PCD in *B. cinerea* hyphae

**Figure 7:**
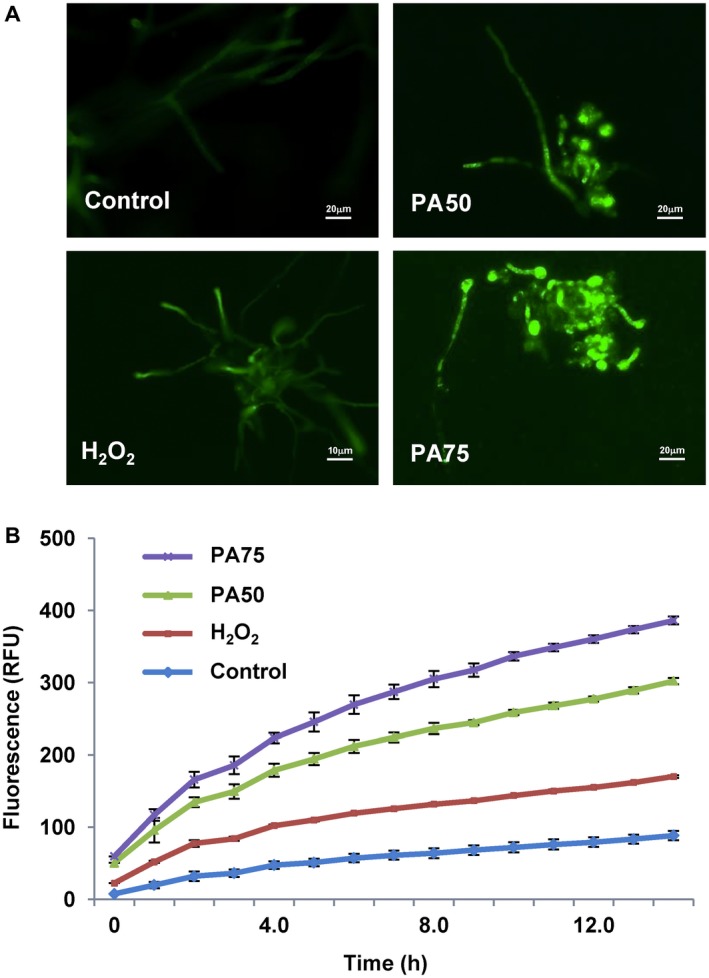
*Pseudozyma aphidis* activates reactive oxygen species (ROS) accumulation in *Botrytis cinerea* hyphae. (A) Intracellular ROS levels in *B. cinerea* were detected by staining with DHR123 after exposure to *P. aphidis* (PA) or H_2_O_2_ as a control. Green fluorescence indicates ROS accumulation. (B) Fluorescence was also detected for 14 h using a TECAN reader plate after exposure to 50% (PA50) or 75% (PA75) of *P. aphidis *secretions or 5 mm H_2_O_2_ or water as controls.

**Figure 8:**
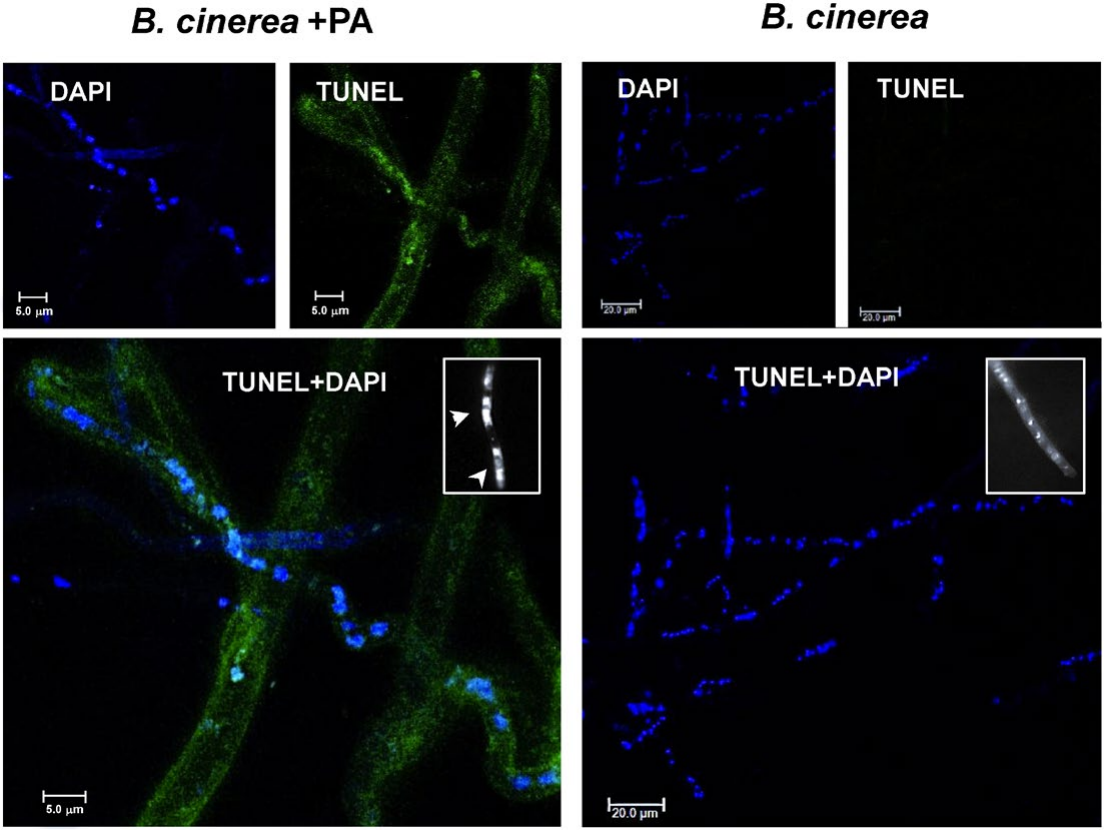
*Pseudozyma aphidis* induces programmed cell death (PCD) in *Botrytis cinerea*. PCD was detected in *B. cinerea* during the interaction with *P. aphidis* *in vitro* (*B. cinerea* + PA) or *B. cinerea* alone (*B. cinerea*) by terminal deoxynucleotidyl transfererase dUTP nick end labelling (TUNEL) and 4′,6‐diamidino‐2‐phenylindole (DAPI) staining, and confocal laser scanning microscopy. Green fluorescence of the nuclei in these images indicates positive TUNEL staining of nuclei; blue represents stained DNA. The insets on the right side of the merged photographs (TUNEL + DAPI) demonstrate DNA condensations marked with white arrowheads (*B. cinerea* + PA) and intact nuclei (*B. cinerea*).

Intracellular ROS levels increased in *B. cinerea *mycelium after exposure to *P. aphidis* extract in a concentration‐dependent manner (Fig. 7 **)**. Furthermore, terminal deoxynucleotidyl transfererase dUTP nick end labelling (TUNEL) assay and 4′,6‐diamidino‐2‐phenylindole (DAPI) staining revealed the activation of apoptotic‐like cell death in *B. cinerea* hyphae by *P. aphidis* extract (Fig. 8). We also observed chromatin condensation and DNA breaks in *B. cinerea* hyphae as markers of PCD (Fig. 8).

## Discussion

The mechanisms involved in the biological suppression of infection and inoculum potential of *B. cinerea* are numerous and variable, and have been demonstrated in several systems (Elad, 1996). The elucidation of the mechanisms of action of antagonistic strains, such as *P. aphidis*, is useful to increase their efficient use against plant pathogens, and to identify fundamental traits in antagonistic activity that could be applied to other plant pathogens. With this in mind, we detailed the potential interactions and modes of action involved in the biocontrol activity of *P. aphidis* against *B. cinerea*. Such modes of action include antibiosis, competition and resistance induction (Buxdorf *et al.*, 2013a,b), but the underlying molecular mechanisms were never completely studied. As demonstrated in the current work, *P. aphidis *antagonistic activity against *B. cinerea* can be significantly reduced by the presence of an external carbon source (Fig. 2). This suggests that competition for nutrients is another possible mode of action for *P. aphidis*, similar to other yeasts and yeast‐like organisms with biocontrol activity against necrotrophic pathogens (Bencheqroun *et al.*, 2007; Castoria *et al.*, 2001; Filonow *et al.*, 1996; Hu *et al.*, 2017; Janisiewicz *et al.*, 2000; Lima *et al.*, 1997; Liu *et al.*, 2013; Lopes *et al.*, 2015). Alternatively, the addition of nutrients may allow the pathogen to accelerate its growth and overcome the antagonistic effects of *P. aphidis*, such as antibiosis and resistance induction.

The role of *P. aphidis* and the antifungal compounds produced by the studied strain in its antagonistic activity against *B. cinerea* were thoroughly analysed. Visualization of the interactions revealed that *P. aphidis* triggers strong inhibition of the phytopathogenic fungus *B. cinerea* (Fig. 6B), leading to a reduced hyphal network and a stress response, with different morphological effects on the fungal hyphae, such as swelling, branching, curliness and hypervacuolization. Our results confirm that the negative effects on *B. cinerea* hyphal growth are caused by the presence of antifungal compounds produced by *P. aphidis* (Fig. 6A), which may affect fungal colonization and infection ability. Similar negative effects on hyphal growth and branching of the soil‐borne pathogen *Fusarium oxysporum* f. sp. *radices‐lycopersici* have been described after exposure to the antifungal metabolite produced by the bacterial biocontrol agent* Pseudomonas* *chlororaphis* during their interaction (Bolwerk *et al.*, 2003; Calderón *et al.*, 2014).

Furthermore, *B. cinerea* cells exposed to *P. aphidis* secretions accumulated ROS (Fig. 7 **)** and showed hallmarks of apoptosis (Fig. 8), indicating the involvement of an antibiotic mechanism with PCD activation. Previous studies on *Aspergillus nidulans* have shown that the accumulation of intracellular ROS correlates with apoptosis in the pathogen mycelium (Cheng *et al.*, 2003; Leiter *et al.*, 2005). Observation of the biological agent *Trichoderma pseudokoningii* also demonstrated the ability of antimicrobial compounds to induce ROS accumulation and to activate PCD in *Fusarium oxysporum* cells (Shi *et al.*, 2012). Another study demonstrated that the biocontrol agent *Trichoderma atroviride* also secretes metabolites that activate the plant defence response, such as the induction of ROS accumulation and activation of PCD in the plant host cell (Navazio *et al.*, 2007). These results suggest that the antibiosis mechanism of biocontrol can act at bitrophic levels—on the pathogen and plant host—to control disease. Here, conductivity assays performed with *P. aphidis* did not reveal any disruption of, or ion leakage from, host cells after its application on cucumber plants (Fig. S1, see Supporting Information), but we cannot rule out the possibility that *P. aphidis* secretions contain effectors that might be involved in fungal–fungal or plant–fungal interactions, as suggested recently by Laur *et al.* (2018). The nature of the antifungal compounds produced by *P. aphidis* remains unknown; therefore, we cannot determine whether one or more compounds are involved in the inhibition process during the antagonistic activity, or whether there are other endogenous changes related to antifungal compound production that also participate in the stress response of *B. cinerea* to the presence of *P. aphidis*. We can speculate that several compounds, some with amphiphilic character (Harris R, Gafni A and Levy M, unpublished data), are involved in *P. aphidis*–pathogen–plant interactions, as *P. aphidis* can inhibit both bacterial and fungal pathogens and can also induce plant resistance (Barda *et al.*, 2014; Buxdorf *et al.*, 2013a,b; Gafni *et al.*, 2015). We could not detect any *P. aphidis* parasitism or penetration of *B. cinerea* hyphae in this study, or in previous research using dual culture (Buxdorf *et al.*, 2013a). Interestingly, we observed that *P. aphidis* cells mostly attach to *B. cinerea* hyphae (Fig. 5) during the interaction, and we also detected lytic enzyme activity; these are frequent phenomena of parasitism, but can also be related to the antibiosis activity of this strain, helping *P. aphidis* utilize the nutrients from dead hyphae. Attachment to *B. cinerea* hyphae has been reported for other antagonistic yeasts as well, enabling an increase in biocontrol efficacy without penetration (Allen *et al.*, 2004; Li *et al.*, 2016; Wisniewski *et al.*, 1991). Previously, we have described *P. aphidis* parasitization of *Podosphaera xanthii*: one mode of action, combined with antibiosis activity, consisted of coiling around the pathogen hyphae; again, in this case, we could not see any penetration by the biocontrol agent (Gafni *et al.*, 2015). We can speculate that the attachment action of *P. aphidis* increases the efficacy of its other modes of action, i.e. antibiosis and competition for space and nutrients. The attachment might also support the efficacy of PCD activation by antibiotics, together with the secreted lytic enzymes that contribute to the utilization of nutrients from the pathogen’s dead tissue. The inability to visualize any damage to healthy plant tissue by colonizing or persistent *P. aphidis* (Buxdorf *et al.*, 2013a) suggests that the demonstrated cellulase activity is also connected to the yeast’s ability to utilize nutrients from dead plant tissue.

In conclusion, *P. aphidis* secretions affect *B. cinerea* hyphal morphology and activate ROS production and PCD in fungal mycelium cells. Furthermore, our current data support the notion that competition for nutrients and attachment are additional modes of action of *P. aphidis* against *B. cinerea*.

## Experimental Procedures

### Microorganism and culture conditions

*Pseudozyma aphidis* isolate L12 (Israel) was maintained on potato dextrose agar (PDA; Difco, Bordeaux, France) at 25 °C and transferred weekly to fresh medium. For liquid cultures, *P. aphidis* was grown in PDB (Difco, Bordeaux, France) or MS (Sigma‐Aldrich, St. Louis, Missouri, USA) basal medium when stated for 2–5 days at 25 °C on a rotary shaker at 150 rpm. *Botrytis cinerea* isolate B05.10 was also grown on PDA plates at 22 °C under 12 h of daily illumination. *Pseudozyma aphidis* isolate DSM 70725 was obtained from DSMZ (German Collection of Microorganisms and Cell Cultures, Brauschweig, Germany), and isolates IHEM 18822 and CBS 517.83 were from the Fungal Biodiversity Centre (Utrecht, the Netherlands).

### Persistence and colonization

To perform colonization and persistence assays of *P. aphidis* on plants, 2‐week‐old cucumber plants (*Cucumis sativus* cv. Beit alpha) were grown at 25 °C and 40% relative humidity in a glasshouse. Cucumber plants were sprayed with a GFP‐labelled *P. aphidis *(Gafni *et al.*, 2015) spore suspension (10^8^ spore/mL) and incubated for up to 18 days at 25 °C and 95% relative humidity with 16 h of daylight. Control plants were sprayed with distilled water. Leaves from at least three plants per treatment (*P. aphidis* or distilled water) were taken at 12 and 18 days post‐inoculation for analysis. *Pseudozyma aphidis* cells located on inoculated leaves were considered to be persistent, and those present on new leaves were considered to be colonizers of the assayed *P. aphidis*. Sections (1 cm^2^) of leaves with colonizing or persistent *P. aphidis* were placed directly onto a glass slide with a drop of water and observed by confocal microscopy (LSM‐510, Zeiss, Oberkochen, Germany) using filter sets that monitor GFP (excitation, 488 nm; emission, 501–540 nm).

Cell counts were also determined for persistent and colonized leaf samples. Briefly, leaf samples were weighed and homogenized in a laboratory blender for 1 min with 5 mL of saline solution (0.85%). The resulting suspensions were serially diluted using saline solution and plated on PDA medium supplemented with hygromycin (100 μg/mL). Then, counts of colonies with the appropriate characteristics were determined after 2 days of incubation at 25 °C. Experiments were performed at least twice.

### Competition for nutrients

To assess competition for nutrients as an antagonistic mode of action of *P. aphidis*, an experiment was carried out as described previously (Castoria *et al*., 2001). Briefly, tomato fruit were surface disinfected with 1% (v/v) commercial sodium hypochlorite for 2 min and then rinsed with sterile water. Tomatoes were wounded by 200‐μL Eppendorf tips (Zhang *et al.*, 2015), and then subjected to four different treatments: (i) *P. aphidis* (10 μL of 10^8^ spore/mL); (ii) *B. cinerea* (5 μL of 10^4^ spore/mL); (iii) *P. aphidis* + *B. cinerea*; and (iv) *P. aphidis* + *B. cinerea* + sugar solution (sugar).

To prepare the sugar solution for the inoculation of tomato wounds during the competition experiment, *P. aphidis* carbon assimilation requirements were determined by an ID 32 C kit (BioMerieux, Marcy‐l'Etoile, France) according to the manufacturer’s instructions. *Pseudozyma aphidis* growth was estimated visually and scored (+, positive; W, weakly positive; –, negative). The best carbon sources for the promotion of *P. aphidis* growth were chosen and a final concentration of 1 g/L sugar mix solution was prepared using d‐galactose (5.55 mM), sucrose (2.92 mM), l‐arabinose (6.66 mM), inositol (5.55 mM) and d‐glucose (5.55 mM).

Wounds were treated as follows: 10 μL of *P. aphidis* spore suspension were added to each wound and, after 2 h at room temperature, the wounds were inoculated with 5 μL of *B. cinerea* spore suspension. When sugar solution was also used in the treatment, 10 μL of sugar solution were added to each wound together with the *B. cinerea* inoculation (Castoria *et al.*, 2001). Three replicates of four tomato fruits were performed for each treatment in each experiment. Fruits were kept for 6 days at 25 °C in the dark and 95% relative humidity before recording the percentage of infected wounds. Experiments were repeated twice.

### *In vitro* interaction between *P. aphidis* and *B. cinerea*

To study the interactions between *P. aphidis* and *B. cinerea* *in vitro*, a dual‐culture assay was carried out (Monteiro *et al*., 2014). Plugs (5 mm^2^) from PDA medium were taken from the edge of actively growing colonies of fresh fungal and yeast cultures and placed on the surface of the PDA plate at opposite sides of the plate. The plates were incubated at 25 °C for 7 days; mycelial samples from the interaction region were then collected and examined by DIC microscopy (Nikon Eclipse 80i, Tokyo, Japan) and captured using a Nikon DS‐QiMc camera.

### *In vitro* effects of *P. aphidis* secretions on *B. cinerea*

To study the effects of *P. aphidis* secretions on *B. cinerea*, *P. aphidis* was grown on a PDA plate and incubated at 25 °C for 5 days. *Pseudozyma aphidis* plates were then flipped over and the bottom side was used for inhibition assays with *P. aphidis*‐secreted antibiotics (Buxdorf *et al.*, 2013a). A sterile glass slide was placed on the bottom side of the PDA plate and covered with a thin layer (2–3 mm) of PDA medium. A 0.6‐cm‐diameter agar plug of *B. cinerea* mycelium from a 5‐day‐old fungal culture was placed in the centre of the glass slide, and then incubated for 3 days at 25 °C. PDA plates without *P. aphidis *secretions were used as a negative control. At this point, the glass slides were aseptically removed from the agar plate using a scalpel. Then, a coverslip was placed over and it was observed by DIC microscopy. Figures were captured with a Nikon DS‐QiMc camera and analysed by NIS Elements BR 3.10 software.

### Enzymatic assays

A 2‐day‐old culture of *P. aphidis* was inoculated on the specified agar plates, and incubated at 25 °C. Protease activity was studied by growing *P. aphidis* on 10% dry milk agar plates for 10 days and observing the clearance zone around the colonies. Caseinase activity was studied by growing the isolates on 10% casein agar plates for 5 days. Lipase activity was examined by inoculating the isolates on agar plates with Tween 80 (2% v/v) for 10 days. Cellulase activity was studied on carboxymethylcellulose (10% w/v) agar plates for 7 days following the method of Hankin and Anagnostakis (1977).

To analyse chitinase expression, *P. aphidis* at an optical density at 600 nm (OD_600_) of 0.1 was used for inoculation in 100 mL of different media: (i) MS medium with sucrose (2.92 mM; 1 g/L); (ii) MS medium with sucrose and colloidal chitin (4.92 mM; 1 g/L); and (iii) PDB medium. Cultures were incubated with agitation (150 rpm) at 30 °C. Samples were then taken at 8, 10, 12 and 15 days post‐inoculation for RNA extraction. Total RNA was extracted from all samples and chitinase expression was assayed by quantitative reverse transcription‐polymerase chain reaction (qRT‐PCR) as described later.

### *Botrytis cinerea* cell wall extraction and chitinase expression assay

*Botrytis cinerea* was grown overnight in PDB culture. Washed mycelia were subjected to two cycles of sonication (3 min) and centrifugation (5 min, 2000 ***g***). The preparation was washed six times with water, and then homogenized in 1 : 1 chloroform–methanol (v/v) for 3 min at top speed, and centrifuged. The sediment was taken up in acetone, air dried and resuspended in 10 mm sodium phosphate buffer, pH 6.4, at 2.5 mg/mL (Boller *et al.*, 1983).

For the chitinase expression assay, *P. aphidis* L12 at OD_600_ = 0.1 was used for the inoculation of 100 mL of different media: (i) MS medium without sucrose; (ii) 100 mL of MS medium without sucrose and 4 mL (10 mg) of *B. cinerea* cell wall suspension; (iii) MS medium with sucrose (2.92 mM; 1 g/L); (iv) MS medium with sucrose and colloidal chitin (4.92 mM; 1 g/L); and (v) PDB medium. Cultures were incubated for 10 days on a shaker at 150 rpm and 30 °C. Total RNA was extracted from all samples and chitinase expression was assayed by qRT‐PCR.

### RNA extraction and qRT‐PCR

Total RNA was extracted using TRI Reagent^®^ RNA Isolation Reagent and a Plant/Fungi Total RNA Purification Kit (Norgen Biotek Corp., Thorod, Ontario, Canada), followed by treatment with a Turbo DNA‐Free™ Kit (Ambion, Thermo Fisher Scientific, Waltham, MA, USA). The integrity of the RNA sample was assessed by agarose gel electrophoresis and subsequently used for RT‐PCR experiments. RT‐PCR was performed using a high‐capacity cDNA Reverse Transcription Kit (Applied Biosystems, Foster City, MA, USA). Then, to determine chitinase expression, qRT‐PCR was performed using a SYBR Green Core Kit (Eurogentec, Liege, Belgium) according to the manufacturer’s protocol. The qRT‐PCR primers used for these experiments were as follows: forward, 5′‐TCCTTGCTCGTATGTCTTGC‐3′; reverse, 5′‐AGTACGCAGCAGGCTTGG‐3′. These primers were designed specifically for the *P. aphidis* L12 chitinase gene and did not amplify the *B. cinerea* chitinase gene. qRT‐PCR analysis was carried out following the ΔΔCT method.

### Apoptosis assays and staining procedures

Apoptotic‐like cell death was determined by measurement of chromatin condensation, the number of DNA strand breaks and the accumulation of ROS—criteria that are commonly used to determine apoptotic PCD in fungi (Semighini and Harris, 2010; Sharon *et al.*, 2009). Chromatin condensation was detected following nuclear staining with DAPI, as described previously (Barhoom and Sharon, 2007). Samples were visualized by fluorescence microscopy using a DAPI filter. DNA strand breaks were detected by TUNEL assay using the In Situ Cell Death Detection kit (Roche Applied Science, Basel, Switzerland), as described previously (Shlezinger *et al.*, 2011), with some modifications. Briefly, plugs (5 mm^2^) of *B. cinerea*, previously exposed to *P. aphidis* as described above, were fixed with 4% paraformaldehyde, digested with lysing enzyme from *Trichoderma harzianum* (Sigma‐Aldrich, St. Louis, Missouri, USA), rinsed twice with phosphate‐buffered saline (PBS), incubated with 100 μL of TUNEL reaction mixture for 70 min at 37 °C, and then rinsed twice with PBS. Samples were examined under a confocal microscope with argon ion and two He‐Ne lasers. For a positive control, we followed the manufacturer’s manual and used DNase treatment (Roche Applied Science; Fig. S2, see Supporting Information).

### ROS accumulation

Intracellular ROS levels were detected by staining with dihydrorhodamine 123 (DHR123, Sigma). *Pseudozyma aphidis* was grown in PDB for 2 days at 28 ^o^C; cells were then spun down and discarded and the growth medium was filtered through a 0.4‐μm filter. *Botrytis cinerea* spore suspension (10^5^ spores/mL) was grown in a 48‐well plate containing PDB medium or PDB supplemented with *P. aphidis* secretions (50% and 75% of *P. aphidis* growth medium) or H_2_O_2_ (5 mm) for 14 h at 25 °C with agitation. Following incubation, DHR123 was added to a final concentration of 6 μm to each well and incubated for 1 h at 25 °C with agitation. We detected rhodamine fluorescence resulting from oxidized DHR123 by excitation at 485 nm, and measured the emitted light at 535 nm under a confocal microscope with argon ion and two He‐Ne lasers, or in a plate reader (Infinite F200, Tecan, Zurich, Switzerland). The fluorescence was recorded (four reads per well in triplicate per sample) and registered for every well (48‐well plate, 300 μL per well) every hour for 14 h. For microscopy, *B. cinerea* hyphae from each well were placed on a glass slide and excess PDB medium was removed with a pipette.

## Author Contributions

C.E.C. and M.L. designed the experiments. C.E.C., R.H., N.R. and D.V.‐C. performed the experiments. C.E.C. and M.L. analysed the results and wrote the manuscript. All authors read and approved the final manuscript.

## Supporting information


**Fig. S1** Conductivity assays in cucumber seedlings. Two‐week‐old cucumber seedlings were sprayed with water (Control) or *Pseudozyma aphidis* (PA) at 24 h before inoculation with *Botrytis cinerea* (*B. cinerea* and PA + *B. cinerea*). Samples from all treatments were taken every 24 h for conductivity assays.

**Fig. S2  **Programmed cell death (PCD) activation by DNase. PCD was activated in *Botrytis cinerea* after DNase treatment and detected by terminal deoxynucleotidyl transfererase dUTP nick end labelling (TUNEL) assay and 4′,6‐diamidino‐2‐phenylindole (DAPI) staining, and confocal laser scanning microscopy. Green fluorescence of the nuclei indicates positive TUNEL staining of nuclei; blue represents stained DNA.
