# Supplementary figures and images for: Pseudozyma aphidis activates reactive oxygen species production, programmed cell death and morphological alterations in the necrotrophic fungus Botrytis cinerea
Source: Mol Plant Pathol. 2019 Feb 18;20(4):562–74. doi: 10.1111/mpp.12775 (PMC6637909; doi:10.1111/mpp.12775)

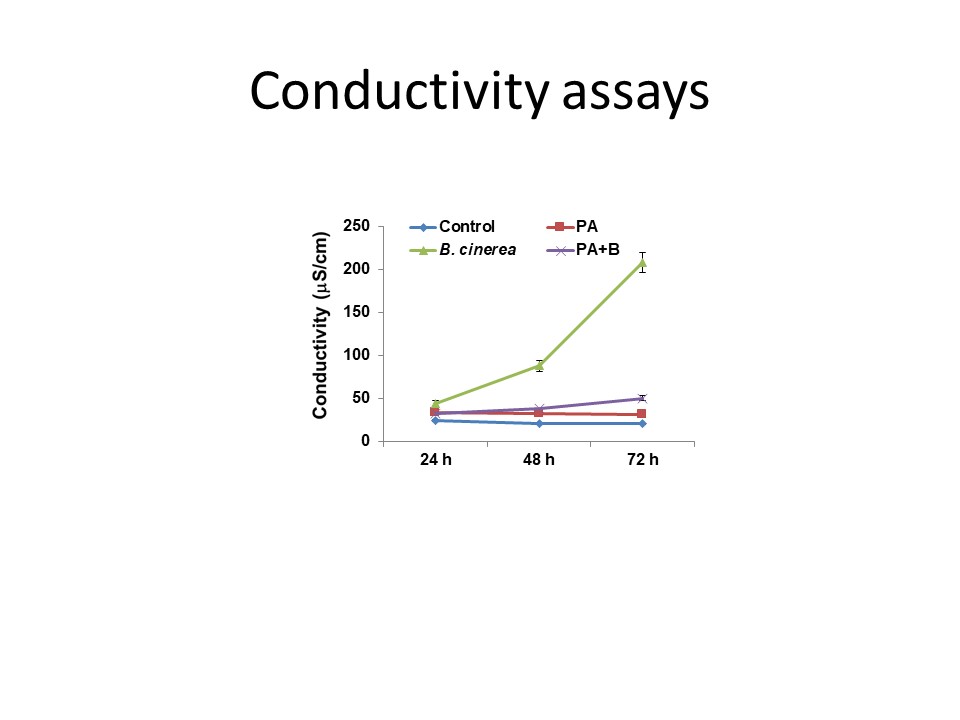

Supplement: Supplementary file 1 — Fig. S1 Conductivity assays in cucumber seedlings. Two‐week‐old cucumber seedlings were sprayed with water (Control) or Pseudozyma aphidis (PA) at 24 h before inoculation with Botrytis cinerea (B. cinerea and PA + B. cinerea). Samples from all treatments were taken every 24 h for conductivity assays. [file MPP-20-562-s001.JPG]

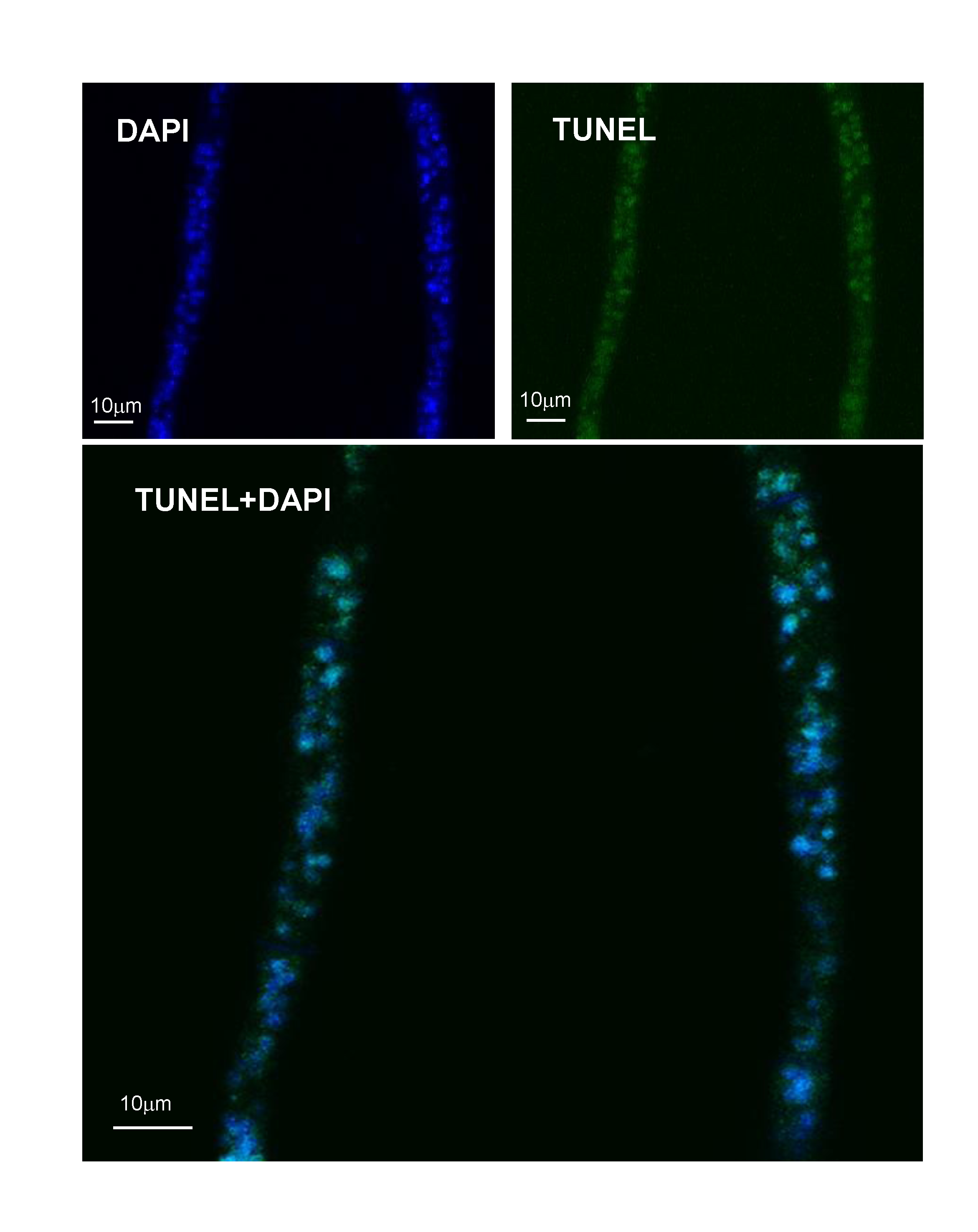

Supplement: Supplementary file 2 — Fig. S2 Programmed cell death (PCD) activation by DNase. PCD was activated in Botrytis cinerea after DNase treatment and detected by terminal deoxynucleotidyl transfererase dUTP nick end labelling (TUNEL) assay and 4′,6‐diamidino‐2‐phenylindole (DAPI) staining, and confocal laser scanning microscopy. Green fluorescence of the nuclei indicates positive TUNEL staining of nuclei; blue represents stained DNA. [file MPP-20-562-s002.tif]
